# Association between perfluoroalkyl acids and kidney function in a cross-sectional study of adolescents

**DOI:** 10.1186/s12940-015-0077-9

**Published:** 2015-11-21

**Authors:** Anglina Kataria, Howard Trachtman, Laura Malaga-Dieguez, Leonardo Trasande

**Affiliations:** Department of Pediatrics, New York University School of Medicine, 227 East 30th St, Room 735, New York, NY 10016 USA; Department of Environmental Medicine, New York University School of Medicine, New York, NY USA; Department of Population Health, New York University School of Medicine, New York, NY USA; New York University Wagner School of Public Service, New York, NY USA; Department of Nutrition, Food Studies, and Public Health, New York University Steinhardt School of Culture, Education, and Human Development, New York, NY USA; New York University Global Institute of Public Health, New York, NY USA

**Keywords:** Perfluoroalkyl acids, eGFR, Uric acid, Adolescents

## Abstract

**Background:**

Perfluoroalkyl acids are synthetic compounds widely used in industrial and commercial applications. Laboratory studies suggest that these persistent and bioaccumulative chemicals produce oxidant stress and damage glomerular endothelial cells, raising concern regarding the impact of these compounds on renal function.

**Methods:**

We performed cross-sectional analyses of data 1960 participants aged 12–19 years of the 2003–2010 National Health and Nutrition Examination Surveys. PFAA exposure was assessed using levels of perfluorooctane sulfonic acid (PFOS), perfluorooctanoic acid (PFOA), perfluorononanoic acid, and perfluorohexane sulfonic acid. Primary study outcomes were estimated glomerular filtration rate (eGFR) and serum uric acid.

**Results:**

While adjusting for demographics, cotinine, prehypertension, insulin resistance, body mass index, and hypercholesterolemia, adolescents in the highest PFOA and PFOS quartile had a lower eGFR, 6.84 mL/min/1.73 m^2^ (95 % CI: 2.19 to 11.48) and 9.69 mL/min/1.73 m^2^ (95 % CI: -4.59 to 14.78), respectively, compared to the lowest quartile. Highest PFOA and PFOS quartiles were also associated with 0.21 mg/dL (95 % CI: 0.056 to 0.37) and 0.19 mg/dL (95 % CI: 0.032 to 0.34) increases in uric acid, respectively.

**Conclusions:**

PFAAs are associated with a reduction in kidney function and increased uric acid levels in otherwise healthy adolescents. Reverse causation and residual confounding could explain the results. Our study results confirm and amplify previous findings, though longitudinal studies examining prenatal and childhood biomarkers in relationship with robust measures of childhood renal function are needed.

## Background

Perfluoroalkyl acids (PFAAs) are synthetic organic compounds with a totally fluorinated carbon chain of varying length and a negatively charged functional group, such as carboxylic or sulfonic acid [1]. This imparts high stability and thermal resistance to these compounds. PFAAs have found wide utility when polymerized in stain-resistant sprays for carpets and upholstery, fire-retarding foams, and nonstick cooking surfaces and food packaging, such as microwave popcorn bags and pizza packaging [2]. National biomonitoring surveys reveal that >98 % of the US population have detectable levels of PFAAs in the blood including: perfluorooctane sulfonic acid (PFOS), perfluorooctanoic acid (PFOA), perfluorohexane sulfonate (PFHxS) and perfluorononanoic acid (PFNA) [3].

In cell culture studies, exposure of microvascular endothelial cells to PFOS induces endothelial permeability through increased production of reactive oxidative species at concentrations relevant to human exposures [4]. This is important given that endothelial cells are vital to structure and function of the glomeruli in the kidney. Furthermore, endothelial permeability plays a critical role in ischemic renal injury [5]. The effect of PFOA, PFOS, PFNA and PFHxS on the kidneys is further magnified by the fact that these compounds are well absorbed orally but have poor renal elimination rates and half-lives in humans of 2.3-8.5 years [6]. Children and adolescents are uniquely vulnerable to PFOA and PFOS as they have greater food consumption per pound body weight [7]. PFAAs are present in human breast milk, and serum levels in children are generally higher than in adults [6, 8]. Childhood exposure may present the additional risk of disrupting cardiovascular and renal physiologic functions, and so vulnerability may be greater than in adults.

Cross-sectional studies have associated PFAA biomarkers with reduced renal function. In one recent large, community-based study of residents living near a fluoropolymer production facility which resulted in contamination of their drinking water with elevated PFOA, serum PFOA, PFOS, PFNA and PFHxS concentrations were inversely correlated with estimated glomerular filtration rate (eGFR) in otherwise healthy children and adolescents [9]. A study by Shankar et al revealed similar findings in adults [10]. The study also found that PFOA and PFOS were associated with increased odds of chronic kidney disease (CKD), defined as eGFR <60 ml/min/1.73 m^2^ [10].

Several cross-sectional epidemiological studies in adults and children have also shown a positive association between PFOA and uric acid (UA) [10–13], though one failed to detect such an association [14]. This is relevant since hyperuricemia has long been thought to be an important marker of renal disease. Moreover, growing evidence suggests that hyperuricemia may be part of the causal pathway in the development of hypertension. Numerous clinical studies have shown that elevated UA levels are associated with increased risk of hypertension, independent of other risk factors [15–18]. UA levels >5.5 mg/dL were observed in 90 % of adolescents with essential hypertension, whereas UA levels were significantly lower in controls and teens with either white-coat or secondary hypertension [16]. Finally, hyperuricemia is an independent risk factor for disease progression in pediatric patients with CKD [19].

However, studies to date have not explored the association between low grade exposure to perfluoroalkyl chemicals in the range commonly experienced by children/adolescents and kidney function. Therefore, we examined the association of PFAAs with kidney function i.e., eGFR and uric acid in a nationally representative sample of US adolescents. We sought to test the hypothesis that higher levels of exposure to PFAAs would be associated with a decrement in kidney function and an increment in serum uric acid concentration.

## Methods

The current study is based on eight years of data (2003–2010) of 12–19 year olds enrolled in the National Health and Nutrition Examination Survey (NHANES), a continuous, multicomponent, nationally representative survey of the non-institutionalized US population administered by the National Center for Health Statistics (NCHS) of the Centers for Disease Control and Prevention (CDC). Data from questionnaire, laboratory, diet, and physical examination components were used in the present analysis, for which data are available in biennial groupings. NHANES oversamples low-income persons, African-Americans, and Mexican-Americans in order to provide stable estimates of these groups. For participants <18 years of age, informed assent was obtained from the participants and informed consent was obtained from the parents or guardians. The survey also includes biomonitoring for perfluoroalkyl chemicals in a random one-third subsample of participants by CDC’s National Center for Environmental Health.

### Exposure measurements

Perfluoroalkyl chemicals were measured in serum using automated solid-phase extraction coupled to isotope dilution high-performance liquid-chromatography-tandem mass spectrometry (details summarized elsewhere [20]). Although our study examined all twelve PFAAs surveyed by NHANES, we primarily focused on PFOA, PFOS, PFNA and PFHxS, which were detected in over 97 % of participants. Values below the limit of detection were reported as the limit of detection divided by square root of 2.

### Measures of outcome

Serum creatinine (Cr) was measured using the modified kinetic Jaffe reaction on two different instruments over the study period: the Beckman Coulter Synchron LX20 (Beckman Coulter, Fullerton, California) in NHANES 2003–2007 [21], and the Beckman Coulter UniCel_®_ DxC800 in NHANES 2008–10. A crossover study performed on a subsample during the instrumentation change, from the Beckman Synchron LX-20 to the Beckman Coulter UniCel_®_ DxC800, revealed the 2007 serum Cr values were slightly higher than the 2008 values. The 2003–2007 Cr values were therefore reduced by 0.08 mg/dL ensuring comparability in the results over the entire study period [22]. In addition, CDC regularly cross-references results to a “gold standard” reference as recommended by the National Kidney Disease Education Program [23]. A comparison of the mean Cr levels of NHANES 2005–06 revealed significant differences between the two methods, requiring additional calibration of the uncorrected values by the following equation: [24]

$$ \mathrm{Corrected}\kern0ex {\mathrm{Cr}}_{\left(2005\hbox{-} 2006\right)}=\hbox{-} 0.016+0.978\ast \kern0ex \left(\mathrm{NHANES}\kern0ex \mathrm{uncalibrated}\kern0ex \mathrm{serum}\kern0ex \mathrm{C}\mathrm{r}\kern0ex \left(\mathrm{mg}/\mathrm{dL}\right)\right) $$

Because serum Cr was measured using the Jaffe reaction during the entire study period, we used the original Schwartz formula, which has been validated as a measure of kidney function in adolescents, to calculate eGFR (mL/min/1.73 m^2^) [25].

UA was measured using the Beckman Synchron LX20 system in NHANES 2003–2007 and Beckman Coulter UniCel_®_ DxC800 in NHANES 2008–2010. Detailed laboratory procedures for the other analytes including cholesterol and cotinine are available on the CDC website [26, 27]. Although measurements were conducted on different instrumentation, CDC has not recommended any corrections. To account for possible confounding by instrumentation method, sensitivity analyses reprised all multivariable analyses by including a covariate for NHANES two-year wave of data collection.

### Potential confounders

We included and adjusted for various potential confounders in our multivariable regression models. Height and weight information was collected by trained technicians following standardized procedures. BMI z-scores were derived using CDC 2000 norms, incorporating height, weight and age and values >95^th^ percentile was defined as obese [28].

Blood pressure (BP) was measured by certified examiners 3 times in all children aged 8–19 years after 5 min of resting in a sitting position. In cases with 1 or more incomplete or interrupted measurements, a fourth attempt was made. Averages of systolic (SBP) and diastolic BP (DBP) measurements were calculated for the purpose of generating categorical BP variables. As BP varies widely by age, sex, and height, we calculated systolic and diastolic BP *z*-scores from mixed-effects linear regression models derived using data from 1999–2000 CDC NHANES. We input sex, age, and height *z*-scores derived from CDC norms to compute expected SBP and DBP [28]. BP *z*-scores were then calculated from the measured BP using the formula *z* = (*x*−μ)/σ, where *x* is the measured BP, μ is the 50^th^ percentile BP, and σ is derived from the same NHANES data [29]. We categorized BP outcomes as present or absent prehypertension (BP ≥90th percentile for age/height *z*-score/sex).

Age, Sex, race/ethnicity, caregiver’s level of education and income were assessed using a questionnaire. Race/ethnicity was categorized into Mexican American, other Hispanic, Non- Hispanic white, non-Hispanic black, and other, based on self-report by 17- to 19- year-olds and caregiver report in 12–16-year-olds. Poverty to income ratio (PIR), calculated by dividing family income by the federal poverty level for family size, was categorized into quartiles. Pregnancy status was assessed through questionnaire and urine pregnancy status; pregnant participants were excluded from our study.

Other measures came from laboratory assessments. Insulin resistance was included in the analyses, as studies have shown insulin resistance to be associated with hypertension and higher UA in children [30]. To assess insulin resistance, we calculated homeostatic model assessment of insulin resistance (HOMA-IR) by multiplying fasting insulin and glucose divided by 22.5. Insulin resistance was analyzed as categorical outcome, using the cut point of 4.39, which is more than 2 SD above the mean HOMA-IR for normal-weight adolescents with normal fasting glucose in NHANES 1999–2002 [31]. Since exposure to tobacco smoke is thought to be a risk factor for kidney disease in adolescents, serum cotinine was also included in the analyses [32]. Cotinine levels were categorized into low (<0.15 ng/mL), medium (≥0.15 ng/mL and >2.0 ng/mL) and high (≥2.0 ng/mL) [33]. Total cholesterol was considered as a potential covariate as lowering cholesterol has been shown to reduce the rate of kidney function loss [34]. Total cholesterol was categorized into low or high groups using the cut-point of 170 mg/dL, based on published guidelines [35].

To maximize sample size in multivariable analysis, missing categories were created for all potential confounders except high cholesterol and BMI category (both n = 1). Less than 10 % of values were missing for any confounding variable, except in case of insulin resistance category, which had 51 % of values missing.

### Statistical analysis

Univariable, bivariate and appropriately weighted multivariable analyses were conducted in a fashion that reflects the complex survey sampling design, using Stata 13.0 (College Station, TX) survey (‘svy’) commands. Bivariate analyses were performed for the sum of all 12 PFAAs to explore potential confounding in PFAA-outcome relationships.

Substantial (>40 %) non-detectable concentrations were identified for 8 of the 12 PFAA. Given the substantial frequency of non-detectable concentrations for the other PFAAs, our analyses focused on PFOA, PFOS, PFHxS and PFNA in the population with available eGFR data. Outcome variables eGFR and UA were analyzed as continuous variables for multivariable analyses. To further confirm the findings, eGFR and UA were categorized into quartile for ordered logistic regression, which is used to predict the odds of categorical severity of the abnormality based on the values of the predictor variables.

Multivariable linear regressions were performed to examine the association between increasing PFAA quartiles and differences in eGFR and UA, adjusting for race/ethnicity, sex, poverty-income ratio, caregiver education, serum cotinine, prehypertension, insulin resistance, BMI, and hypercholesterolemia categories.

We performed sensitivity analyses by adding an NHANES wave covariate to account for confounding by UA measurement method. Finally, given that PFAAs are potential endocrine disruptors [36], we also conducted sex-stratified analyses to assess potential interactions. Because hypercholesterolemia may be an intermediate factor in the causal pathway between PFAAs exposure and renal dysfunction, sensitivity analyses also reprised models without adjustment for cholesterol. Sensitivity analyses also examined addition of continuous age, and substitution of BMI Z-score for categorical body mass. Change of regression coefficients by >10 % or attenuation statistical significance was used in these sensitivity analyses as criteria for suggesting modification of effects.

Finally, we identified in our statistical analyses that PFAA concentrations are strongly correlated with each other. Upon identifying associations of PFOS and PFOA with primary outcomes, in an effort to delineate which of the two were driving effects on renal function, we performed multivariable regressions that simultaneously examined PFOS and PFOA.

## Results

During the study period, 7,168 children and adolescents participated. There were 1961 non-pregnant participants for whom serum perfluoroalkyl concentrations were available. We excluded one participant who had eGFR <60 mL/min/1.73 m^2^, resulting in 1960 participants, with 54.3 % male (Table 1). Mean Cr, eGFR and UA were 0.74 ± 0.17 mg/dL, 144.0 ± 27.6 mL/min/1.73 m^2^ and 5.07 ± 1.19 mg/dL, respectively.

**Table 1:** Adolescents with serum perfluoroalkyl acid measurements, NHANES 2003-2010

	*N* = 1960
Characteristics	Result
Sex, n (%)	
Male	1064 (54.3 %)
Female	896 (45.7 %)
Mean Age ± SD	15.5 ± 2.3
Race, n (%)	
Mexican American	589 (30 %)
Other Hispanics	139 (7.1 %)
Non-Hispanic white	546 (27.9 %)
Non-Hispanic black	594 (30.3 %)
Other race	92 (4.7 %)
Poverty to income ratio, n (%)	
First Quartile (<0.78)	423 (21.6 %)
Second quartile (0.78 to1.45)	449 (22.9 %)
Third Quartile (1.46 to 2.92)	467 (23.8 %)
Fourth Quartile (>2.92)	495 (25.3 %)
Missing	126 (6.4 %)
Parent/Caregiver Education Level, n (%)	
Less than 9th grade	262 (13.4 %)
9-12th grade	372 (19.0 %)
High school Grad/GED	459 (23.4 %)
College or AA degree	522 (26.6 %)
College or greater	259 (13.2 %)
Unknown	86 (4.4 %)
Serum Cotinine, n (%)	
< 0.015 ng/mL	312 (15.9 %)
0.015–1.9 ng/mL	1284 (65.5 %)
At least 2.0 ng/mL	364 (18.6 %)
High Cholesterol, n (%)	647 (33.1 %)
Obese, n (%)	429 (21.9 %)
Insulin resistant	195 (20.3 %)
BP >90th percentile	151 (7.93 %)
Median Creatinine, mg/dL (IQR)	0.72 (0.62, 0.86)
Median eGFR, min/mL/1.73 m^2^ (IQR)	140.4 (125.1, 158.8)
Mean Uric Acid, mg/dL (IQR)	5.0 (4.2, 5.8)

*IQR* interquartile range, *eGFR* estimated glomerular filtration rate

Univariate analyses of renal outcomes against quartiled PFAAs revealed substantial differences among those more highly exposed, with strongest associations for PFOS. Compared with the lowest PFOS levels, all three higher quartiles had lower eGFR with 6.31 (95 % CI:−11.1 to−1.49), 6.56 (95 % CI:−12.0 to−1.06) and 9.76 (95 % CI:−15.1 to−4.44) mL/min/1.73 m^2^ decrements. Adolescents with the highest PFOA concentrations had 4.37 mL/min/1.73 m^2^ lower eGFR (95 % CI:−9.15 to 0.40). No significant associations with PFHxS or PFNA were identified.

Bivariate analyses revealed substantial differences in PFAAs, eGFR and uric acid by social, behavioral and other characteristics (Appendix Tables 3 and 4), supporting potential confounding and inclusion of covariates in multivariable models. Non-Hispanic white and non-Hispanic black participants had significantly higher mean creatinine and lower eGFR. Obesity was associated with significantly lower creatinine, higher eGFR and higher uric acid levels. Insulin resistance was also associated with lower creatinine, higher eGFR and higher uric acid levels. High levels of cotinine (>2 ng/mL) were associated with significantly increased creatinine and uric acid and decreased eGFR. Prehypertension and high total cholesterol were found to be associated with significantly increased uric acid level but not with creatinine and eGFR. Participants with caregivers who had 9–12^th^ grade education, high school/graduate equivalency diploma or college/Associate’s degree had significantly higher creatinine and lower eGFR than those with less than 9^th^ grade or college and greater education.

Bivariate analyses of PFAAs revealed males to have higher total PFAA than female. Non-Hispanic whites and non-Hispanic blacks had total PFAA levels in the 4^th^ quartile more frequently than other race/ethnicities. Highest total PFAA quartile was also correlated with increasing quartiles of cotinine levels, worsening poverty-income ratio and higher education level of parent/caregivers. On the other hand, obesity and insulin resistance were associated with lower total PFAA quartiles. Age, high total cholesterol and prehypertension did not have significant associations with total PFAA levels.

Upon control for potential confounders, multivariable linear regression revealed adolescents in the highest quartile of PFOA to have 6.61 mL/min/1.73 m^2^ lower eGFR than adolescents in the lowest quartile (95 % CI: -11.39 to−1.83, Table 2). Similarly, when examining the relationship between PFOS and eGFR, the multivariable regression revealed a negative correlation such that all higher quartiles of PFOS are associated with a statistically significant decrease in eGFR quartile, compared to the lowest quartile. Most notably, quartile 4 of PFOS was associated with a 9.47 mL/min/1.73 m^2^ lower eGFR (95 % CI:−14.68 to−4.25). Results for eGFR and UA were non-significant for PFHxS and PFNA.

**Table 2:** Multivariable regression of perfluoroalkyl acids with outcome measure of kidney function

	eGFR (min/mL/1.73 m^2^)	Uric Acid (mg/dL)
	Increment (95 % CI)	Increment (95 % CI)
Perfluorooctanoic acid (PFOA)		
Quartile 1 (<2.5 ng/mL)	Reference	Reference
Quartile 2 (2.5–3.5 ng/mL)	−2.63 (−7.07 to 1.81)	0.17 (−0.033 to 0.37)
Quartile 3 (3.5–4.7 ng/mL	−5.42 (−11.46 to 0.61)	0.13 (−0.030 to 0.28)
Quartile 4 (≥4.7 ng/mL)	−6.61 (−11.39 to−1.83)**	0.21 (0.056 to 0.37)**
Perfluorooctane sulfonic acid (PFOS)		
Quartile 1 (<7.9 ng/mL)	Reference	Reference
Quartile 2 (7.9–12.8 ng/mL)	−5.24 (−9.75 to−0.73)*	0.095 (−0.081 to 0.27)
Quartile 3 (12.8 19.4 ng/mL)	−7.21 (−12.21 to−2.21)**	0.046 (−0.10 to 0.19)
Quartile 4 (≥19.4 ng/mL)	−9.47 (−14.68 to−4.25)***	0.19 (0.032 to 0.34)*
Perfluorononanoic Acid (PFNA)		
Quartile 1 (<0.7 ng/mL)	Reference	Reference
Quartile 2 (0.7–1.0 ng/mL)	1.24 (−4.29 to 6.78)	0.013 (−0.17 to 0.20)
Quartile 3 (1.0–1.5 ng/mL)	2.76 (−1.68 to 7.20)	−0.035 (−0.20 to 0.13)
Quartile 4 (≥1.5 ng/mL)	−1.06 (−5.49 to 3.37)	0.12 (−0.051 to 0.29)
Perfluorohexane Sulfonic acid (PFHxS)		
Quartile 1 (<1 ng/mL)	Reference	Reference
Quartile 2 (1–2 ng/mL)	1.37 (−3.59 to 6.34)	0.040 (−0.14 to 0.22)
Quartile 3 (2–3.95 ng/mL)	1.85 (−3.36 to 7.05)	0.053 (−0.13 to 0.23)
Quartile 4 (≥4 ng/mL)	−0.32 (−4.44 to 3.81)	−0.054 (−0.22 to 0.11)

All models adjust for Sex, poverty-income ratio, caregiver education, serum cotinine, prehypertension, insulin resistance, Body Mass Index, hypercholesterolemia and race/ethnicity categories

Conversion factors for units: serum creatinine in mg/dL to μmol/L, ×88.4; serum uric acid in mg/dL to μmol/L, x59.48

*eGFR* estimated glomerular filtration rate

* *p* < 0.05 ** *p* < 0.01 *** *p* < 0.001

The highest PFOA and PFOS quartile were also associated with a 0.21 mg/dL (p = 0.009) and 0.19 mg/dL (p = 0.019) increase in UA levels compared with lowest quartile, respectively (Table 2). Multivariable analyses failed to show any significant associations of PFHxS and PFNA with any of the outcome variables.

Ordered logistic regression revealed that increasing PFOS quartiles were associated with significant decrease in eGFR, with adolescents in highest PFOS quartile having a 1.96-fold higher risk of having eGFR in the lower quartiles (95 % CI: 1.37, 2.86; p < 0.001). Adolescents in the highest PFOA and PFOS quartiles had significantly increased risk of having UA in higher quartiles, compared to those in the lower PFOA/PFOS quartiles (Appendix Table 6).

Sex-stratified regression models did not alter associations of PFAA with eGFR or UA measures, and addition of NHANES two-year wave covariate failed to change regression coefficients by >10 % or attenuate statistical significance. Sensitivity analyses also reprised models without adjustment for cholesterol (Appendix Table 5), and failed to find a change in regression coefficients by >10 % or attenuate statistical significance. Sensitivity analyses failed to find significant alteration of associations resulting from addition of continuous age, or substitution of BMI Z-score for categorical body mass.

Finally, PFOA was substantially correlated with PFOS (r = 0.60). Simultaneous examination of PFOA and PFOS revealed significant decrements in eGFR and increases in uric acid in association with PFOA but not PFOS. Compared with the lowest quartile, adolescents with higher levels of PFOA had 3.90 (95 % CI:−8.04 to 0.24), 5.84 (95 % CI:−11.56 to−0.12) and 5.77 (95 % CI:−11.01 to 0.54) mL/min/1.73 m^2^ lower eGFR. Compared with the lowest quartile, adolescents with higher levels of PFOA had 0.20 (95 % CI: 0.02 to 0.39), 0.15 (95 % CI:−0.006 to 0.32) and 0.23 (95 % CI: 0.02 to 0.43) mg/dL higher uric acid.

## Discussion

In a representative sample of 1960 US adolescents, we found higher concentrations of PFOA and PFOS, but not PFHxS or PFNA in serum to be associated with a decrements in eGFR and increases in UA, independent of numerous confounders including demographic features, other environmental exposures and lifestyle variables. All the statistical analyses, including evaluating exposures as categorical variables by quartiles and ordered logistic regression, indicate that graded exposure to these chemicals is associated with reductions in eGFR and UA. These results are consistent with previous cross-sectional studies, including a community-based study of adolescents exposed to PFOA-contaminated water, and cross-sectional study of adult NHANES participants [9–11, 13]. The smaller decrement in eGFR (<1 mL/min/1.73 m^2^) in a much more highly exposed sample compared with our estimates (7−10 mL/min/1.73 m^2^) suggests the possibility of steeper dose-response relationship in the lower ranges of exposure, which has also been identified for lead [37], PFAAs [38] and other environmental chemicals though we cannot exclude residual confounding. A steeper dose-response curve at lower exposure ranges for various endpoints has also been found for PFAAs (including PFOA and PFOS) for several endpoints including liver enzymes, cholesterol, and UA [12, 39, 40]. In addition, the relationship between PFAA exposure and eGFR may be more apparent in adolescents with normal kidney function compared to adults who may have subclinical renal disease.

Evidence from animal studies suggests the association between PFAAs and renal function is biologically plausible. The finding of renal hypertrophy and histopathological alterations indicate that cell proliferation and injury occur in rats exposed to PFOA and PFOS, likely induced by reactive oxidative species [4, 41]. These rat models exhibited generally higher concentrations of PFOS in all organs compared to PFOA, likely due to slower elimination of PFOS. This may explain the relatively stronger associations with PFOS observed in our study [41]. One preclinical study has suggested that early life exposure to PFAA could produce elevated blood pressure [42].

Increases in serum UA concentration could potentially be part of the causal pathway between PFAAs and eGFR given that it is a potent pro-oxidant in the intracellular environment and induces oxidative stress and endothelial dysfunction. The net effect of high UA levels may be development of hypertension, interstitial fibrosis, glomerulosclerosis and eventual kidney failure [43]. The mechanisms underlying the association between hyperuricemia and PFAAs are not clear. PFAAs may compete with anion transporters that are normally responsible for UA secretion, ultimately resulting in decreased UA excretion [44]. Alternatively, since UA is renally cleared, higher UA levels could also be the consequence of decreased kidney function due to PFAA exposure. Further mechanistic studies are needed to clarify the biological association between UA and PFAAs.

While all our participants had normal renal function, the 7−10 mL/min/1.73 m^2^ decrease in eGFR observed with higher levels of the compounds is not insignificant. Moreover, studies of essential hypertension in children have shown that for each 1 mg/dl change in UA, there is an associated average increase of 14 mmHg in systolic blood pressure and 7 mmHg in diastolic blood pressure [16]. Slight increases in serum uric acid concentration within the normal range, comparable to changes noted in our study, have been associated with a larger decline in GFR and a higher incidence of CKD over a 2-year period in a community-based adult Japanese cohort [45]. The increase in UA that we documented in association with PFAA exposure may lead to a change in blood pressure that may be important on a population level. Given the prolonged half-life of these chemicals, levels of PFAAs in these adolescents are likely to persist into adulthood, and so the impact of these compounds on renal function is likely magnified as the chemicals induce oxidant stress over an extended period of time. As these children age, their renal functional reserve diminishes, and so in the context of exposure to other potential environmental toxins or conditions, the rate of their kidney dysfunction may be accelerated, increasing the risk of CKD.

Reverse causality is a major limitation of our study, and could represent an alternate explanation of our findings, in that serum PFAAs could be higher simply because of reduced excretion. While we note the C8 panel finding that there is not a probable link between PFOA exposure and kidney disease [46], the unique vulnerability of children to PFAAs has not been studied longitudinally, let alone in the lower ranges of exposure in whom steeper dose-response relationships are noted. Could a 5−10 % difference in renal function (a 7-10 mL/min/1.73 m^2^ decrement from a median of 140.4) produce a twofold increase in PFAA? While this is a possibility, the present evidence cannot provide definitive proof that it does. Indeed, the serum concentration of most molecules that are cleared by the kidney does not begin to rise until the GFR falls below 60–90 ml/min/1.73 m^2^.

In one recent large, community based study of residents living near a production facility exposed to contaminated water, serum PFAA levels were inversely correlated with eGFR in otherwise healthy children and adolescents [9]. The authors of this paper argue that curve attenuation may indicate reverse causation. However, curve attenuation at higher dose has characterized the findings of many known outcome relationships with toxins (or drugs) that interact with nuclear receptors [47], and could explain the differential findings between the NHANES population and the population living near a production facility. PFOS concentrations are affected by human enterohepatic factors [48, 49], yet Watkins et al found PFOS to be strongly associated with eGFR.

Han et al note that organic anion transporters in the proximal tubule cells result in the secretion and then reabsorption of these compounds [50]. Some use these data together with null findings about antecedent PFAA exposure described by Watkins et al [9] to postulate that the site of renal dysfunction is tubular rather than glomerular. We have made no a priori assumption on the primary site of injury within the kidney. Based on what is known about the ability of PFAAs to induce oxidant stress, both glomeruli and tubules are equally plausible as targets of PFAA-induced renal injury and both would result in a decline in GFR. We could not study tubular function in the NHANES sample, and believe that an optimal study would longitudinally measure both glomerular and tubular function, potentially adjusting for the latter. The Watkins study did not adjust for tubular function.

Watkins et al describes a highly exposed sample (median PFOA fourfold higher than the lower margin of the highest quartile in our sample) in which antecedent exposures were modeled from resident address and water measurements, with modest predictive value in children (62 %) [9]. Biomarker data were not available prior to measured renal function. While some might argue that the Watkins et al study proves reverse causation, a single study cannot result in such a conclusion, just as forward causation cannot be determined from a single study. Although our study results are incremental in that they chiefly confirm and amplify previous findings, they support the need for better longitudinal study with serially collected biomarker data.

There are a few other limitations to the study including the cross-sectional nature of this study, which prevents us from inferring causality. Other limitations include lack of data on potential confounders such as diet, family history of kidney disease and Tanner staging, as PFAAs are known to have estrogenic activity [36, 51]. We were also unable to control for other environmental chemicals which are oxidant stressors and have been associated with albuminuria and cardiorenal risks [52–54]. We could not ascertain the degree to which the PFAAs were albumin bound as opposed to freely circulating, which may vary among individuals and contribute to the interindividual differences in renal function associated with PFAAs in this study [55]. While increased binding might mitigate the nephrotoxic effects of PFAAs, reduced protein binding might result in greater uptake by renal cells and exacerbate injury.

We also note that the Schwartz formula is but one measure of glomerular function. We also could not examine the albumin-to-creatinine ratio, because in children, only first morning voids yield valid measurements, and these were only available in NHANES 2009–10. Measurements of Cystatin C have led to development of alternate formulas, and as have other formulas using beta-trace protein [56]. Beyond glomerular function, tubular function could not be assessed in our study.

Our study suggests perfluoroalkyl chemicals present as modifiable risk factors for kidney dysfunction that could be eliminated with a voluntary phase out of the four PFAAs that remains on target to complete a phase-out by 2015 [57]. Given their persistence in the environment, continued formation from precursor compounds, and the potential for continued production by non-participating manufacturers in the U.S. and/or overseas, environmental contamination and human exposure with the potential for health impacts from these PFAAs is expected to continue even after they are phased out, as seen in the case of PFOS. Furthermore, numerous PFAAs continue to be used. A Swedish study of PFAA trends between 1996–2010 confirmed increases in PFHxS (4.3 %/year), but also noted 11 % increases/year of a four-carbon PFAA, perfluoroalkylbutane sulfonate (PFBS), a substitute for PFOS increasingly found in food. [58] These findings suggest that the adverse effects of PFAAs on kidney function in children and adolescents will continue to represent a potential health problem in the coming years. Longitudinal research is therefore highly relevant, both to clarify the observed associations of eGFR and UA with PFAAs, and ongoing efforts to reduce long-term health effects of PFAA exposure.

## Conclusions

In this cross-sectional study of healthy adolescents, we found levels of PFOA and PFOS to be significantly associated with decreased kidney function within the normal range and increased serum UA levels. Prospective studies are needed to confirm the association and understand biological mechanisms underlying this relationship.

## Appendix

**Table 3:** Mean creatinine, eGFR and uric acid, by sample characteristics

	Number	Mean Creatinine	*P*-value	Mean eGFR_CKiD_	*P*-value	Mean eGFR_Schwartz_	*P*-value	Mean Uric Acid	*P*-value
Overall	1961	0.75		95.7		142.9		301.8	
Sex									
Male	1064	0.81	P < 0.001	90.9	P < 0.001	149.7	P < 0.001	333.8	P < 0.001
Female	897	0.68		101.3		134.9		263.7	
Age									
12	233	0.60		110.8		147.5		270.4	P < 0.001
13	249	0.64	P < 0.001	107.0	P < 0.001	163.3	P < 0.001	283.1	
14	258	0.71		98.9		150.9		301.0	
15	227	0.76		93.8		143.4		311.4	
16	246	0.81		90.5		137.0		309.3	
17	244	0.81		89.0		136.4		314.9	
18	267	0.82		87.8		131.9		311.9	
19	237	0.82		88.2		133.0		310.9	
Race/Ethnicity									
Mexican American	589	0.70	Reference	99.9	Reference	147.9	Reference	299.4	Reference
Other Hispanics	139	0.70	P < 0.876	101.1	P < 0.516	151.0	P < 0.224	305.7	P < 0.354
Non-Hispanic white	547	0.77	P < 0.001	95.2	P < 0.001	143.5	P < 0.005	314.0	P < 0.001
Non-Hispanic black	594	0.79	P < 0.001	90.2	P < 0.001	135.2	P < 0.001	292.1	P < 0.073
Other race	92	0.72	P < 0.499	98.4	P < 0.451	144.6	P < 0.279	300.4	P < 0.899
Poverty to Income Ratio									
First Quartile (<0.78)	423	0.75	Reference	95.5	Reference	141.3	Reference	305.2	Reference
Second quartile (0.78 to1.45)	449	0.74	P < 0.591	95.4	P < 0.941	141.6	P < 0.837	295.1	P < 0.035
Third Quartile (>1.46 to 2.92)	468	0.75	P < 0.898	96.1	P < 0.633	144.5	P < 0.079	300.6	P < 0.335
Fourth Quartile (>2.92)	495	0.75	P < 0.590	95.4	P < 0.930	143.7	P < 0.176	306.2	P < 0.825
Missing	126	0.75	P < 0.873	96.2	P < 0.705	143.9	P < 0.341	300.8	P < 0.536
Parent/Caregiver Education Level									
Less than 9th grade	263	0.71	Reference	100.3	Reference	147.5	Reference	299.8	Reference
9-12th grade	372	0.74	P < 0.044	95.4	P < 0.001	142.3	P < 0.019	299.9	P < 0.978
High school Grad/GED	459	0.77	P < 0.001	93.9	P < 0.001	141.9	P < 0.008	304.2	P < 0.420
College or AA degree	522	0.76	P < 0.001	94.8	P < 0.001	141.9	P < 0.007	303.1	P < 0.537
College or greater	259	0.74	P < 0.048	96.3	P < 0.015	142.2	P < 0.028	297.9	P < 0.766
Unknown	86	0.75	P < 0.076	95.3	P < 0.032	144.9	P < 0.432	306.7	P < 0.428
Serum Cotinine									
< 0.015 ng/mL	312	0.69	Reference	102.2	Reference	148.5	Reference	284.1	Reference
0.015-1.9 ng/mL	1284	0.74	P < 0.001	96.6	P < 0.001	143.9	P < 0.007	299.0	P < 0.001
≥ 2.0 ng/mL	365	0.85	P < 0.001	86.9	P < 0.001	134.8	P < 0.001	326.7	P < 0.001
High Cholesterol									
Total Cholesterol < 170 mg/dL	1312	0.74	P < 0.310	96.1	P < 0.115	144.1	P < 0.005	298.1	P < 0.001
Total Cholesterol ≥ 170 mg/dL	648	0.75		94.7		140.4		309.0	
Obesity Status									
Not Obese	1531	0.75	P < 0.016	94.8	P < 0.001	141.6	P < 0.001	290.9	P < 0.00
Obese	429	0.73		98.7		147.4		340.3	
Insulin Status									
HOMA-IR <4.39	767	0.76	P < 0.009	95.2	P < 0.001	142.4	P < 0.001	295.4	P < 0.001
HOMA-IR ≥4.39	195	0.71		101.1		150.3		337.4	
Blood Pressure Status									
Blood Pressure <90th Percentile	1752	0.75	P < 0.503	95.4	P < 0.353	142.5	P < 0.272	300.2	P < 0.006
Blood Pressure ≥90th Percentile	151	0.74		96.9		145.0		316.5

**Table 4:** Bivariate analysis of total serum of perfluoroalkyl acids by demographics, anthropometric and clinical characteristics

	Total Perfluoroalkyl Acids				
*N* = 1961	Quartile 1 (<15.2 ng/mL)	Quartile 2 (15.2–22.1 ng/mL)	Quartile 3 (22.1–32.1 ng/mL)	Quartile 4 (≥32.1 ng/mL)	P-value
	n, (%)				
Overall	490 (25.0 %)	487 (24.8 %)	494 (25.2 %)	490 (25.0 %)	
Sex					
Male	211 (19.8 %)	234 (22.0 %)	294 (27.6 %)	325 (30.6 %)	P < 0.001
Female	279 (31.1 %)	253 (28.2 %)	200 (22.3 %)	165 (18.4 %)	
Race					
Mexican American	205 (34.8 %)	168 (28.5 %)	121 (20.5 %)	95 (16.1 %)	P < 0.001
Other Hispanics	68 (48.9 %)	33 (23.7 %)	26 (18.7 %)	12 (8.6 %)	
Non-Hispanic white	84 (15.4 %)	128 (23.4 %)	160 (29.3 %)	175 (32.0 %)	
Non-Hispanic black	107 (18.0 %)	136 (22.9 %)	169 (28.5 %)	182 (30.6 %)	
Other race	26 (28.3 %)	22 (23.9 %)	18 (19.6 %)	26 (28.3 %)	
Poverty to Income Ratio					
First Quartile (<0.78)	138 (32.6 %)	101 (23.9 %)	98 (23.2 %)	86 (20.3 %)	P < 0.001
Second quartile (0.78 to1.45)	133 (29.6 %)	104 (23.2 %)	116 (25.8 %)	96 (21.4 %)	
Third Quartile (>1.46 to 2.92)	99 (21.25)	120 (25.6 %)	126 (26.9 %)	123 (26.3 %)	
Fourth Quartile (>2.92)	82 (16.6 %)	130 (26.3 %)	125 (25.3 %)	158 (31.9 %)	
Missing	38 (30.2 %)	32 (25.4 %)	29 (23.0 %)	27 (21.4 %)	
Parent/Caregiver Education					
Less than 9^th^ grade	112 (42.6 %)	77 (29.3 %)	49 (18.6 %)	25 (9.5 %)	P < 0.001
9-12^th^ grade	94 (25.3 %)	98 (26.3 %)	110 (29.6 %)	70 (18.8 %)	
High school Graduate or GED	105 (22.9 %)	109 (23.7 %)	118 (25.7 %)	127 (27.7 %)	
College or AA degree	101 (19.4 %)	106 (20.3 %)	142 (27.2 %)	173 (33.1 %)	
College graduate or greater	54 (20.9 %)	72 (27.8 %)	56 (21.6 %)	77 (29.7 %)	
Unknown	24 (27.9 %)	25 (29.1 %)	19 (22.1 %)	18 (20.1 %)	
Serum Cotinine					
< 0.015 ng/mL	103 (33.0 %)	76 (24.4 %)	68 (21.8 %)	65 (20.8 %)	P < 0.001
0.015–1.9 ng/mL	336 (26.2 %)	312 (24.3 %)	320 (24.9 %)	316 (24.6 %)	
≥ 2.0 ng/mL	51 (14.0 %)	99 (27.1 %)	106 (29 %)	109 (29.9 %)	
High Cholesterol					
Total Cholesterol < 170 mg/dL	345 (26.3 %)	331 (25.2 %)	327 (24.9 %)	309 (23.6 %)	P < 0.097
Total Cholesterol ≥ 170 mg/dL	145 (22.4 %)	156 (24.1 %)	166 (25.6 %)	181 (27.9 %)	
Obesity Status					
Not Obese	365 (23.8 %)	366 (23.9 %)	399 (20.1 %)	401 (26.25)	P < 0.007
Obese	125 (29.1 %)	120 (28 %)	95 (22.1 %)	89 (20.8 %)	
Insulin Status					
HOMA-IR <4.39	191 (24.9 %)	178 (23.2 %)	208 (27.1 %)	190 (24.8 %)	P < 0.001
HOMA-IR ≥4.39	70 (35.9 %)	58 (29.7 %)	36 (18.5 %)	31 (15.9 %)	
Blood Pressure Status					
Blood Pressure <90th Percentile	437 (24.9 %)	433 (24.7 %)	441 (25.2 %)	441 (25.2 %)	P < 0.674
Blood Pressure ≥90th Percentile	37 (24.5 %)	44 (29.1 %)	35 (23.2 %)	35 (23.2 %)

**Table 5:** Multivariable regression of perfluoroalkyl acids with outcome measure of kidney function unadjusted for cholesterol

	Creatinine (mg/dL)	eGFR (min/mL/1.73 m^2^)	Uric Acid (mg/dL)
Perfluorooctanoic acid (PFOA)			
Quartile 1 (<2.5 ng/mL)	Reference	Reference	Reference
Quartile 2 (2.5–3.5 ng/mL)	0.007 (−0.012 to 0.027)	−2.81 (−7.16 to 1.53)	0.17 (–0.028 to 0.37)
Quartile 3 (3.5–4.7 ng/mL	0.021 (−0.008 to 0.050)	−5.50 (−11.50 to 0.50)	0.13 (−0.030 to 0.29)
Quartile 4 (≥4.7 ng/mL)	0.029 (0.004 to 0.054)*	−6.84 (−11.48 to−2.19)**	0.23 (0.071 to 0.39)**
Perfluorooctane sulfonic acid (PFOS)			
Quartile 1 (<7.9 ng/mL)	Reference	Reference	Reference
Quartile 2 (7.9–12.8 ng/mL)	0.021 (−0.007 to 0.049)	−5.39 (−9.87 to−0.92)*	0.11 (−0.063 to 0.29)
Quartile 3 (12.8 19.4 ng/mL)	0.038 (0.008 to 0.068)*	−7.25 (−12.25 to−2.25)**	0.045 (−0.10 to 0.19)
Quartile 4 (≥19.4 ng/mL)	0.040 (0.010 to 0.071)**	−9.69 (−14.78 to−4.59)***	0.20 (0.041 to 0.35)*
Perfluorononanoic Acid (PFNA)			
Quartile 1 (<0.7 ng/mL)	Reference	Reference	Reference
Quartile 2 (0.7–1.0 ng/mL)	−0.003 (−0.035 to 0.029)	1.25 (−4.30 to 6.80)	0.011 (−0.17 to 0.19)
Quartile 3 (1.0–1.5 ng/mL)	−0.018 (−0.046 to 0.009)	2.59 (−1.89 to 7.08)	−0.027 (−0.19 to 0.14)
Quartile 4 (≥1.5 ng/mL)	0.0034 (−0.24 to 0.031)	−1.2 (−5.62 to 3.22)	0.13 (−0.035 to 0.30)
Perfluorohexane Sulfonic acid (PFHxS)			
Quartile 1 (<1 ng/mL)	Reference	Reference	Reference
Quartile 2 (1–2 ng/mL)	−0.000 (−0.029 to 0.029)	1.27 (−3.67 to 6.21)	0.053 (−0.12 to 0.23)
Quartile 3 (2–3.95 ng/mL)	−0.007 (−0.037 to 0.023)	1.81 (−3.40 to 7.03)	0.052 (−0.13 to 0.23)
Quartile 4 (≥4 ng/mL)	−0.008 (−0.036 to 0.020)	−0.31 (−4.39 to 3.77)	−0.057 (–0.23 to 0.11)

All models adjust for gender, poverty-income ratio, caregiver education, serum cotinine, prehypertension, insulin resistance, Body Mass Index, and race/ethnicity categories

Conversion factors for units: serum creatinine in mg/dL to μmol/L, ×88.4; serum uric acid in mg/dL to μmol/L, x59.48

*eGFR* estimated glomerular filtration rate

* *p* < 0.05 ** *p* < 0.01 *** *p* < 0.001

**Table 6:** Ordered logistic regression analysis of kidney function outcome measures against perfluoroalkyl acids

	Odds Ratio for Higher Creatinine Quartile	Odds ratio for Lower eGFR Quartiles	Odds Ratio for Higher Uric Acid Quartiles
PFOA			
Quartile 1 (<2.5 ng/mL)	Reference	Reference	Reference
Quartile 2 (2.5–3.5 ng/mL)	1.19 (0.91, 1.56)	1.16 (0.85, 1.59)	1.30 (0.89, 1,89)
Quartile 3 (3.5–4.7 ng/mL	1.41 (0.95, 2.09)	1.54 (1.02, 2.33)*	1.24 (0.88, 1.74)
Quartile 4 (≥4.7 ng/mL)	1.59 (1.13, 2.25)**	1.69 (1.19, 2.38)**	1.49 (1.08, 2.06)*
PFOS			
Quartile 1 (<7.9 ng/mL)	Reference	Reference	Reference
Quartile 2 (7.9–12.8 ng/mL)	1.36 (0.93, 1.99)	1.37 (1.03, 1.82)*	1.15 (0.83, 1.58)
Quartile 3 (12.8 19.4 ng/mL)	1.64 (1.12, 2.41)*	1.54 (1.09, 2.17)*	1.08 (0.78, 1.51)
Quartile 4 (≥19.4 ng/mL)	1.70 (1.12, 2.59)*	1.96 (1.37, 2.86)***	1.46 (1.07, 2.01)*
PFNA			
Quartile 1 (<0.7 ng/mL)	Reference	Reference	Reference
Quartile 2 (0.7–1.0 ng/mL)	1.11 (0.74, 1.66)	0.95 (0.671, 1.35)	1.16 (0.76, 1.79)
Quartile 3 (1.0–1.5 ng/mL)	0.87 (0.62, 1.21)	0.78 (0.55, 1.11)	1.02 (0.70, 1.49)
Quartile 4 (≥1.5 ng/mL)	1.28 (0.90, 1.81)	1.20 (0.85, 1.69)	1.39 (0.96, 2.01)
PFHxS			
Quartile 1 (<1 ng/mL)	Reference	Reference	Reference
Quartile 2 (1–2 ng/mL)	0.99 (0.66, 1.48)	0.99 (0.66, 1.49)	1.09 (0.79, 1.50)
Quartile 3 (2–3.95 ng/mL)	0.94 (0.64, 1.40)	0.88 (0.62, 1.25)	1.11 (0.77, 1.61)
Quartile 4 (≥4 ng/mL)	0.91 (0.63, 1.33)	1.09 (0.77, 1.54)	0.95 (0.69, 1.31)

All models control for Sex, poverty-income ratio, caregiver education, serum cotinine, prehypertension, insulin resistance, Body Mass Index, hypercholesterolemia and race/ethnicity categories. These ordered logistic regressions estimate one equation over all levels of the outcome variable and the coefficients shown per quartile of exposure represent the odds of being in the higher versus the lower quartiles of the outcome variable (or in the lower versus the higher quartiles in the case of eGFR)

*PFOA* perfluorononanoic acid, *PFOS* perfluorooctane sulfonic acid, *PFNA* perfluorononanoic acid, *PFHxS* perfluorohexane sulfonic acid, *eGFR* estimated glomerular filtration rate

* *p* < 0.05 ** *p* < 0.01 *** *p* < 0.001

## Abbreviations

BP: Blood pressure
CDC: Centers for Disease Control and Prevention
CKD: Chronic Kidney Disease
Cr: Creatinine
eGFR: Estimated glomerular filtration rate
NCHS: National Center for Health Statistics
NHANES: National Health and Nutrition Examination Survey
PFAAs: perfluoroalkyl acids
PFHxS: perfluorohexane sulfonate
PFNA: perfluorononanoic acid
PFOS: perfluorooctane sulfonic acid
PFOA: perfluorooctanoic acid
UA: uric acid

## References

[1] Buck RC, Franklin J, Berger U, Conder JM, Cousins IT, de Voogt P (2011). Perfluoroalkyl and polyfluoroalkyl substances in the environment: terminology, classification, and origins. Integr Environ Assess Manag.

[2] Trudel D, Horowitz L, Wormuth M, Scheringer M, Cousins IT, Hungerbühler K (2008). Estimating Consumer Exposure to PFOS and PFOA. Risk Anal.

[3] Calafat AM, Wong L-Y, Kuklenyik Z, Reidy JA, Needham LL (2007). Polyfluoroalkyl chemicals in the US population: data from the National Health and Nutrition Examination Survey (NHANES) 2003–2004 and comparisons with NHANES 1999–2000. Environ Health Perspect.

[4] Qian Y, Ducatman A, Ward R, Leonard S, Bukowski V, Lan Guo N (2010). Perfluorooctane sulfonate (PFOS) induces reactive oxygen species (ROS) production in human microvascular endothelial cells: role in endothelial permeability. J Toxicol Environ Health A.

[5] Sutton TA, Mang HE, Campos SB, Sandoval RM, Yoder MC, Molitoris BA (2003). Injury of the renal microvascular endothelium alters barrier function after ischemia. Am J Physiol Renal Physiol.

[6] Lau C, Anitole K, Hodes C, Lai D, Pfahles-Hutchens A, Seed J (2007). Perfluoroalkyl acids: a review of monitoring and toxicological findings. Toxicol Sci.

[7] Hoek G, Meliefste K, Cyrys J, Lewne M, Bellander T, Brauer M (2002). Spatial variability of fine particle concentrations in three European areas. Atmospheric Env.

[8] Post GB, Cohn PD, Cooper KR (2012). Perfluorooctanoic acid (PFOA), an emerging drinking water contaminant: a critical review of recent literature. Environ Res.

[9] Watkins DJ, Josson J, Elston B, Bartell SM, Shin HM, Vieira VM (2013). Exposure to perfluoroalkyl acids and markers of kidney function among children and adolescents living near a chemical plant. Environ Health Perspect.

[10] Shankar A, Xiao J, Ducatman A (2011). Perfluoroalkyl chemicals and chronic kidney disease in US adults. Am J Epidemiol.

[11] Geiger SD, Xiao J, Shankar A (2013). Positive Association Between Perfluoroalkyl Chemicals and Hyperuricemia in Children. Am J Epidemiol.

[12] Steenland K, Tinker S, Shankar A, Ducatman A (2010). Association of perfluorooctanoic acid (PFOA) and perfluorooctane sulfonate (PFOS) with uric acid among adults with elevated community exposure to PFOA. Environ Health Perspect.

[13] Gleason JA, Post GB, Fagliano JA (2015). Associations of perfluorinated chemical serum concentrations and biomarkers of liver function and uric acid in the US population (NHANES), 2007-2010. Environ Res.

[14] Lin CY, Lin LY, Wen TW, Lien GW, Chien KL, Hsu SH (2013). Association between levels of serum perfluorooctane sulfate and carotid artery intima-media thickness in adolescents and young adults. Int J Cardiol.

[15] Alper AB, Chen W, Yau L, Srinivasan SR, Berenson GS, Hamm LL (2005). Childhood uric acid predicts adult blood pressure: the Bogalusa Heart Study. Hypertension.

[16] Feig DI, Johnson RJ (2003). Hyperuricemia in childhood primary hypertension. Hypertension.

[17] Sundstrom J, Sullivan L, D'Agostino RB, Levy D, Kannel WB, Vasan RS (2005). Relations of serum uric acid to longitudinal blood pressure tracking and hypertension incidence. Hypertension.

[18] Forman JP, Choi H, Curhan GC (2007). Plasma uric acid level and risk for incident hypertension among men. J Am Soc Nephrol.

[19] Warady BA, Abraham AG, Schwartz GJ, Wong CS, Munoz A, Betoko A (2015). Predictors of Rapid Progression of Glomerular and Nonglomerular Kidney Disease in Children and Adolescents: The Chronic Kidney Disease in Children (CKiD) Cohort. Am J Kidney Dis.

[20] Kuklenyik Z, Reich JA, Tully JS, Needham LL, Calafat AM (2004). Automated solid-phase extraction and measurement of perfluorinated organic acids and amides in human serum and milk. Environ Sci Technol.

[21] National Center for Health Statistics. Laboratory Procedure Manual: Creatinine in Refrigerated Serum: 2003-2004. [ Available at http://www.cdc.gov/nchs/data/nhanes/nhanes_03_04/l40_c_met_creatinine.pdf (Accessed 12 August 2014)].

[22] National Center for Health Statistics. Laboratory Procedure Manual: Creatinine in Refrigerated Serum: 2007-2008. [ Available at http://wwwn.cdc.gov/Nchs/Nhanes/2007-2008/BIOPRO_E.htm (Accessed 12 August 2014)].

[23] National Center for Health Statistics. Laboratory Procedure Manual: Creatinine in Refrigerated Serum: 2005-2006 [ Available at http://www.cdc.gov/nchs/data/nhanes/nhanes_05_06/biopro_d_met_creatinine.pdf (Accessed 12 August 2014)]

[24] Selvin E, Manzi J, Stevens LA, Van Lente F, Lacher DA, Levey AS (2007). Calibration of serum creatinine in the National Health and Nutrition Examination Surveys (NHANES) 1988-1994, 1999-2004. Am J Kidney Dis.

[25] Schwartz GJ, Brion LP, Spitzer A (1987). The use of plasma creatinine concentration for estimating glomerular filtration rate in infants, children, and adolescents. Pediatr Clin North Am.

[26] National Center for Health Statistics. CDC Laboratory Procedure Manual: Uric Acid in Refrigerated Serum 2003-2004. [Available at http://www.cdc.gov/nchs/data/nhanes/nhanes_03_04/l40_c_met_uric_acid.pdf (Accessed 12 August 2014)]

[27] National Center for Health Statistics. CDC Laboratory Procedure Manual: Uric Acid in Refrigerated Serum 2009-2010 [ Available at http://www.cdc.gov/NCHS/data/nhanes/nhanes_09_10/BIOPRO_F_met_uric%20acid.pdf (Accessed 12 August 2014)]

[28] Ogden CL, Kuczmarski RJ, Flegal KM, Mei Z, Guo S, Wei R (2002). Centers for Disease Control and Prevention 2000 growth charts for the United States: improvements to the 1977 National Center for Health Statistics version. Pediatrics.

[29] National High Blood Pressure Education Program Working Group on High Blood Pressure in Children and Adolescents (2004). The fourth report on the diagnosis, evaluation, and treatment of high blood pressure in children and adolescents. Pediatrics.

[30] Viazzi F, Antolini L, Giussani M, Brambilla P, Galbiati S, Mastriani S (2013). Serum uric acid and blood pressure in children at cardiovascular risk. Pediatrics.

[31] Lee JM, Okumura MJ, Davis MM, Herman WH, Gurney JG (2006). Prevalence and determinants of insulin resistance among US adolescents a population-based study. Diabetes Care.

[32] García-Esquinas E, Loeffler LF, Weaver VM, Fadrowski JJ, Navas-Acien A (2013). Kidney Function and Tobacco Smoke Exposure in US Adolescents. Pediatrics.

[33] Wilson K, Finkelstein J, Blumkin A, Best D, Klein J (2011). Micronutrient levels in children exposed to secondhand tobacco smoke. Nicotine Tob Res.

[34] Tonelli M, Isles C, Craven T, Tonkin A, Pfeffer MA, Shepherd J (2005). Effect of Pravastatin on Rate of Kidney Function Loss in People With or at Risk for Coronary Disease. Circulation.

[35] American Academy of Pediatrics (1992). National Cholesterol Education Program: report of the expert panel on blood cholesterol levels in children and adolescents. Pediatrics.

[36] White SS, Fenton SE, Hines EP (2011). Endocrine disrupting properties of perfluorooctanoic acid. J Steroid Biochem Mol Biol.

[37] Lanphear BP, Hornung R, Khoury J, Yolton K, Baghurst P, Bellinger DC (2005). Low-Level Environmental Lead Exposure and Children’s Intellectual Function: An International Pooled Analysis. Environ Health Perspect.

[38] Maisonet M, Näyhä S, Lawlor DA, Marcus M (2015). Prenatal exposures to perfluoroalkyl acids and serum lipids at ages 7 and 15 in females. Environ Int.

[39] Steenland K, Fletcher T, Savitz DA (2010). Epidemiologic evidence on the health effects of perfluorooctanoic acid (PFOA). Environ Health Perspect.

[40] Gallo V, Leonardi G, Genser B, Lopez-Espinosa MJ, Frisbee SJ, Karlsson L (2012). Serum perfluorooctanoate (PFOA) and perfluorooctane sulfonate (PFOS) concentrations and liver function biomarkers in a population with elevated PFOA exposure. Environ Health Perspect.

[41] Cui L, Zhou Q-F, Liao C-Y, Fu J-J, Jiang G-B (2009). Studies on the toxicological effects of PFOA and PFOS on rats using histological observation and chemical analysis. Arch Environ Contam Toxicol.

[42] Rogers JM, Ellis-Hutchings RG, Grey BE, Zucker RM, Norwood J, Grace CE (2014). Elevated blood pressure in offspring of rats exposed to diverse chemicals during pregnancy. Toxicol Sci.

[43] Sanchez-Lozada LG, Lanaspa MA, Cristobal-Garcia M, Garcia-Arroyo F, Soto V, Cruz-Robles D (2012). Uric acid-induced endothelial dysfunction is associated with mitochondrial alterations and decreased intracellular ATP concentrations. Nephron Exp Nephrol.

[44] Eraly SA, Vallon V, Rieg T, Gangoiti JA, Wikoff WR, Siuzdak G (2008). Multiple organic anion transporters contribute to net renal excretion of uric acid. Physiol Genomics.

[45] Kamei K, Konta T, Hirayama A, Suzuki K, Ichikawa K, Fujimoto S (2014). A slight increase within the normal range of serum uric acid and the decline in renal function: associations in a community-based population. Nephrol Dial Transplant.

[46] C8 Science Panel: Probable link evaluation. Available at http://www.c8sciencepanel.org/prob_link.html (Accessed 15 August 2014). 2014.

[47] Stayner L, Steenland K, Dosemeci M, Hertz-Picciotto I (2003). Attenuation of exposure-response curves in occupational cohort studies at high exposure levels. Scand J Work Environ Health.

[48] Genuis SJ (2011). Elimination of persistent toxicants from the human body. Hum Exp Toxicol.

[49] Genuis SJ, Birkholz D, Ralitsch M, Thibault N (2010). Human detoxification of perfluorinated compounds. Public Health.

[50] Han X, Nabb DL, Russell MH, Kennedy GL, Rickard RW (2012). Renal elimination of perfluorocarboxylates (PFCAs). Chem Res Toxicol.

[51] Benninghoff AD, Bisson WH, Koch DC, Ehresman DJ, Kolluri SK, Williams DE (2011). Estrogen-Like Activity of Perfluoroalkyl Acids In Vivo and Interaction with Human and Rainbow Trout Estrogen Receptors In Vitro. Toxicol Sci.

[52] Trasande L, Sathyanarayana S, Trachtman H (2014). Dietary phthalates and low-grade albuminuria in US children and adolescents. Clin J Am Soc Nephrol.

[53] Trasande L, Sathyanarayana S, Spanier AJ, Trachtman H, Attina TM, Urbina EM (2013). Urinary phthalates are associated with higher blood pressure in childhood. J Pediatr.

[54] Trasande L, Attina TM, Trachtman H (2013). Bisphenol A exposure is associated with low-grade urinary albumin excretion in children of the United States. Kidney Int.

[55] Han X, Snow TA, Kemper RA, Jepson GW (2003). Binding of Perfluorooctanoic Acid to Rat and Human Plasma Proteins. Chem Res Toxicol.

[56] White CA, Akbari A, Doucette S, Fergusson D, Hussain N, Dinh L (2009). Estimating GFR using serum beta trace protein: accuracy and validation in kidney transplant and pediatric populations. Kidney Int.

[57] Long-Chain Perfluorinated Chemicals (PFCs) [http://www2.epa.gov/assessing-and-managing-chemicals-under-tsca/long-chain-perfluorinated-chemicals-pfcs]. Accessed 19 November 2015.

[58] Glynn A, Berger U, Bignert A, Ullah S, Aune M, Lignell S (2012). Perfluorinated alkyl acids in blood serum from primiparous women in Sweden: serial sampling during pregnancy and nursing, and temporal trends 1996-2010. Environ Sci Technol.

